# Supplementary material for: Studying item-effect variables and their correlation patterns with multi-construct multi-state models
Source: PLoS One. 2023 Aug 21;18(8):e0288711. doi: 10.1371/journal.pone.0288711 (PMC10441805; doi:10.1371/journal.pone.0288711)
Supplement: S1 Appendix — Appendix A: Overview on recent applications with item-specific method effectsAppendix B: Different scenarios for zero or perfect correlations with item-effect variablesAppendix C: Description of the scalesAppendix D: Path diagrams ○ Path diagrams of the multi-state models of Life Satisfaction○ Path diagram of the common model of Life Satisfaction and Positive AffectAppendix E: Model fit for unidimensional models. (DOCX) [file pone.0288711.s001.docx]

Appendix

**Appendix A: Overview on recent applications with item-specific method effects**

Benefits of item-specific method effects have already been shown for specific constructs (cognitive components of Alzheimer’s disease, children’s inattention symptoms, subjective happiness, life satisfaction) in the empirical studies of Cogo-Moreira et al. (2020), Geiser et al. (2019), Holtmann et al. (2020), and Thielemann et al. (2017). An overview on the scales and applications is shown in Table A1.

**Table A1. Overview of recent studies investigating item-specific method effects.**

| Study / Scale | Scale level | Approach | $N$ | No. items | Mm. oc. |
| --- | --- | --- | --- | --- | --- |
| Cogo-Moreira et al. (2020) |  |  |  |  |  |
| ADAS-Cog  (3 subdomains) |  | item-effect residuals | 341 | 11 ^(a)^ | 4 |
| - Language | categ. |  |  | 3 |  |
| - Praxis | categ. |  |  | 4 |  |
| - Memory | contin. |  |  | 5 |  |
| Geiser et al. (2019) |  |  |  |  |  |
| CADBI (Inattention scale) | contin. | item-effect residuals | 805 | 3^(b)^ | 3 |
| Holtmann et al. (2020) |  |  |  |  |  |
| SWLS (selected items) | categ. | item-effect residuals | 501 | 3 | 4 |
| SHS | categ. |  |  | 3 |  |
| Thielemann et al. (2017) |  |  |  |  |  |
| FPI-R life satisfaction scale | dicho. | item-effect variables | 485 | 12 | 3 |

*Note*. ADAS-Cog = Alzheimer’s Disease Assessment Scale–Cognitive Subscale; CADBI = Child and Adolescent Disruptive Behavior Inventory; SWLS = Satisfaction with Life Scale; SHS = Subjective Happiness Scale; FPI-R = Freiburg Personality Inventory–Revised; categ. = categorical; contin. = continuous; dicho. = dichotomous; Mm.oc. = Number of measurement occasions. (a) some items were included in more than one subdomain. (b) nine items of the inattention subscale were used to generate three parcels.

**Appendix B: Different scenarios for zero or perfect correlations with item-effect variables**

For measuring one construct at different measurement occasions with a multi-item scale, we define latent state variables $\eta_{t}:=\tau_{1t}$ (i.e., the latent ability at a specific time point $t$, measured with the state variable of the first item that is chosen as the reference item) and latent item-effect variables $\delta_{it}:={\tau_{it}-\tau}_{1t}$ (i.e., the latent person-specific difference of using another item $i$ instead of the reference item for measuring the latent construct).

Of interest are the correlations between the latent state variables and item-effect variables, that are, $Cor(\eta_{t},\delta_{it})$for different time points $t$ and items $i$, and among the item-effect variables of different items $i$ and $k,$that are $Cor(\delta_{it},\delta_{kt})$. For describing the meaning of the correlations, we first consider special cases without person-specific differences between the items, but constant differences across all persons. Specifically, either an essentially equivalent measurement model holds (i.e., $\tau_{it}={\nu_{it}+\tau}_{1t}$), where items differ in an intercept $\nu_{it}$, or a congeneric model holds (i.e., $\tau_{it}={\nu_{it}+\lambda_{it}\tau}_{1t}$), where items differ in an intercept $\nu_{it}$ and factor loading $\lambda_{it}$. In these cases, the parameters of the item-effect variables can directly be linked to the intercepts and factor loadings.

Under the assumption of an essentially equivalent measurement model, it can be shown that,

$${E(\delta}_{it})=E\left( {\tau_{it}-\tau}_{1t} \right)=E\left( {\nu_{it}+\tau}_{1t}-\tau_{1t} \right)=\nu_{it},$$

$${Var(\delta}_{it})=Var\left( \tau_{it}-\tau_{1t} \right)=Var\left( \nu_{it}+\tau_{1t}-\tau_{1t} \right)=0,$$

the expected value of the item-effect variables corresponds to the intercept and their variance is zero. Due to a zero variance, all correlations with the item-effect variables are zero, too. Thus, in this case no systematic variance is prevalent and can be explained.

Instead, when a congeneric model holds, then,

$${Var(\delta}_{it})=Var(\tau_{it}-\tau_{1t})=Var\left( \nu_{it}+\lambda_{it}\tau_{1t}-\tau_{1t} \right)=\left( \lambda_{it}-1 \right)^{2}Var\left( \tau_{1t} \right),$$

systematic variation is present in relation to the loading parameter. Yet, this variation can perfectly be explained by the latent state, whereas the covariance is,

$$Cov\left( \eta_{t},\delta_{it} \right)=Cov\left( \tau_{1t},\nu_{it}+\lambda_{it}\tau_{1t}-\tau_{1t} \right)=Cov\left( \tau_{1t},\tau_{1t}(\lambda_{it}-1) \right) =\left( \lambda_{it}-1 \right)Var\left( \tau_{1t} \right),$$

that can be insert in the formula for the correlation,

$$Cor\left( \eta_{t},\delta_{it} \right)=\frac{Cov\left( \eta_{t},\delta_{it} \right)}{\sqrt{Var(\eta_{t})}\sqrt{Var(\delta_{it})}}=\frac{\left( \lambda_{it}-1 \right)Var\left( \tau_{1t} \right)}{\sqrt{Var\left( \tau_{1t} \right)}\sqrt{\left( \lambda_{it}-1 \right)^{2}Var\left( \tau_{1t} \right)}}=\pm1.$$

Note, in cases where the factor loading is negative, the correlation would be negative, too. Similarly, the correlations among item-effect variables of different items $i$ and $k$, for which a congeneric model holds can be derived, with the covariance,

$$Cov\left( \delta_{it},\delta_{kt} \right)=Cov\left( \nu_{it}+\lambda_{it}\tau_{1t}-\tau_{1t},\nu_{kt}+\lambda_{kt}\tau_{1t}-\tau_{1t} \right)=\left( \lambda_{it}-1 \right)\left( \lambda_{kt}-1 \right)Var\left( \tau_{1t} \right),$$

that can be insert in the formula for the correlation,

$$Cor\left( \delta_{it},\delta_{kt} \right)=\frac{Cov\left( \delta_{it},\delta_{kt} \right)}{\sqrt{Var(\delta_{it})}\sqrt{Var(\delta_{kt})}}=\frac{\left( \lambda_{it}-1 \right)\left( \lambda_{kt}-1 \right)Var\left( \tau_{1t} \right)}{\sqrt{\left( \lambda_{it}-1 \right)^{2}Var\left( \tau_{1t} \right)}\sqrt{\left( \lambda_{kt}-1 \right)^{2}Var\left( \tau_{1t} \right)}}=\pm1.$$

Accordingly, in this case, there is one common source for the systematic variation between items, that is the latent state itself, due to different loadings. If one loading is negative, the correlation would be negative, too.

In the described special cases, it would not be necessary to model item-effect variables. Instead, when a congeneric model does not hold, then items differ not only in constant parameters and the item effect variables $\delta_{it}:=\tau_{it}-\tau_{1t}$ cannot be simplified in form of a linear transformation of the latent state variable. This is the more general case, in which various causes for the item-effect variables can be present. The correlations of item-effect variables with the states $Cor\left( \eta_{t},\delta_{it} \right)$ indicate whether the states are a possible explanation for the systematic non-state variance, due to differences in factor loadings or common explanatory variables. Also, the correlations among item-effect variables of different items $Cor\left( \delta_{it},\delta_{kt} \right)$ indicate whether common or comparable causes (e.g., the latent state variables as well as other person characteristics) are plausible. An example for a common cause for item-effect variables of two different items would be a wording effect (i.e., the reference item is a positive statement, but the items $i$and $k$ are negatively formulated). If such a wording effect is the only cause for differences between items, it can be modelled as a common method variable $W=\tau_{it}-\tau_{1t}=\tau_{kt}-\tau_{1t}$, that is similar for both items (or a linear transformation in a more general case). In this case the correlation of the latent state variable with the item-effect variables depends on the correlation with $W$,

$$Cor\left( \eta_{t},\delta_{it} \right)=\frac{Cov\left( \eta_{t},\delta_{it} \right)}{\sqrt{Var(\eta_{t})}\sqrt{Var(\delta_{it})}}=\frac{Cov\left( \eta_{t},W \right)}{\sqrt{Var\left( \tau_{1t} \right)}\sqrt{Var\left( W \right)}}$$

In the most extreme case, the correlation of the latent state variables with the item-effect variables is zero, which is often assumed in residual definitions of method effects. Meaning the method effect and the latent state are distinct variance components that systematically affect the item-responses. Instead, the correlation of the item-effect variables is still perfect in this case,

$$Cor\left( \delta_{it},\delta_{kt} \right)=\frac{Cov\left( \delta_{it},\delta_{kt} \right)}{\sqrt{Var(\delta_{it})}\sqrt{Var(\delta_{kt})}}=\frac{Var(W)}{\sqrt{Var(W)}\sqrt{Var(W)}}=\pm1,$$

Note, if the item-effect variable of one item is a negative transformation of $W$, the correlation would be negative, too. The correlation among item-effect variables reduces in its absolute value, the more distinct the cause for different item-effect variables are. It can be zero in the most extreme case of completely distinct causes for item-effect variables of different items.

**Appendix C: Description of the scales**

The *Satisfaction with Life Scale* contains five items that are rated from “strongly disagree” to “strongly agree” in seven steps. The five items were presented as items 014-018 in the personality questionnaire of the LISS panel and were rated from “strongly disagree” to “strongly agree” in seven steps. Find the items in Table C1.

**Table C1. Satisfaction with Life Scale.**

| Below are five statements with which you may agree or disagree. Using the 1-7 scale below, indicate your agreement with each item by placing the appropriate number on the line preceding that item. Please be open and honest in your responding. | |
| --- | --- |
| 014 | In most ways my life is close to my ideal |
| 015 | The conditions of my life are excellent |
| 016 | I am satisfied with my life |
| 017 | So far I have gotten the important things I want in life |
| 018 | If I could live my life over, I would change almost nothing |
| Response scale: 1 = *strongly disagree*, 2 = *disagree*, 3 = *slightly disagree*, 4 = *neither agree nor disagree*, 5 = *slightly agree*, 6 = *agree*, 7 = *strongly agree*. | |

Positive affect is measured by ten items of the *Positive and Negative Affect Schedule*. The items were shown in the personality questionnaire of the LISS panel as items 146-165 and were rated from “not at all” to “extremely” in seven steps. Find the positive affect items in Table C2.

**Table C2. Positive affect items of the Positive and Negative Affect Schedule.**

| Indicate to what extent you feel, right now, that is, at the present moment… | | | |
| --- | --- | --- | --- |
| 146 | interested? | 157 | alert? |
| 148 | excited? | 159 | inspired? |
| 150 | strong? | 161 | determined? |
| 154 | enthusiastic? | 162 | attentive? |
| 155 | proud? | 164 | active? |
| Response scale: 1 = *not at all*, 7 = *extremely*. | | | |

**Appendix D: Path diagrams**

**Path diagrams of the multi-state models of Life Satisfaction**

For life satisfaction, as well as for positive affect, the multi-state models with and without item effects are compared. See Fig D.1 for the illustrating path diagram of the multi-state model and Fig D.2 for the multi-state model with item effects measuring five latent state variables ${\eta_{LS}}_{t}$ of life satisfaction.


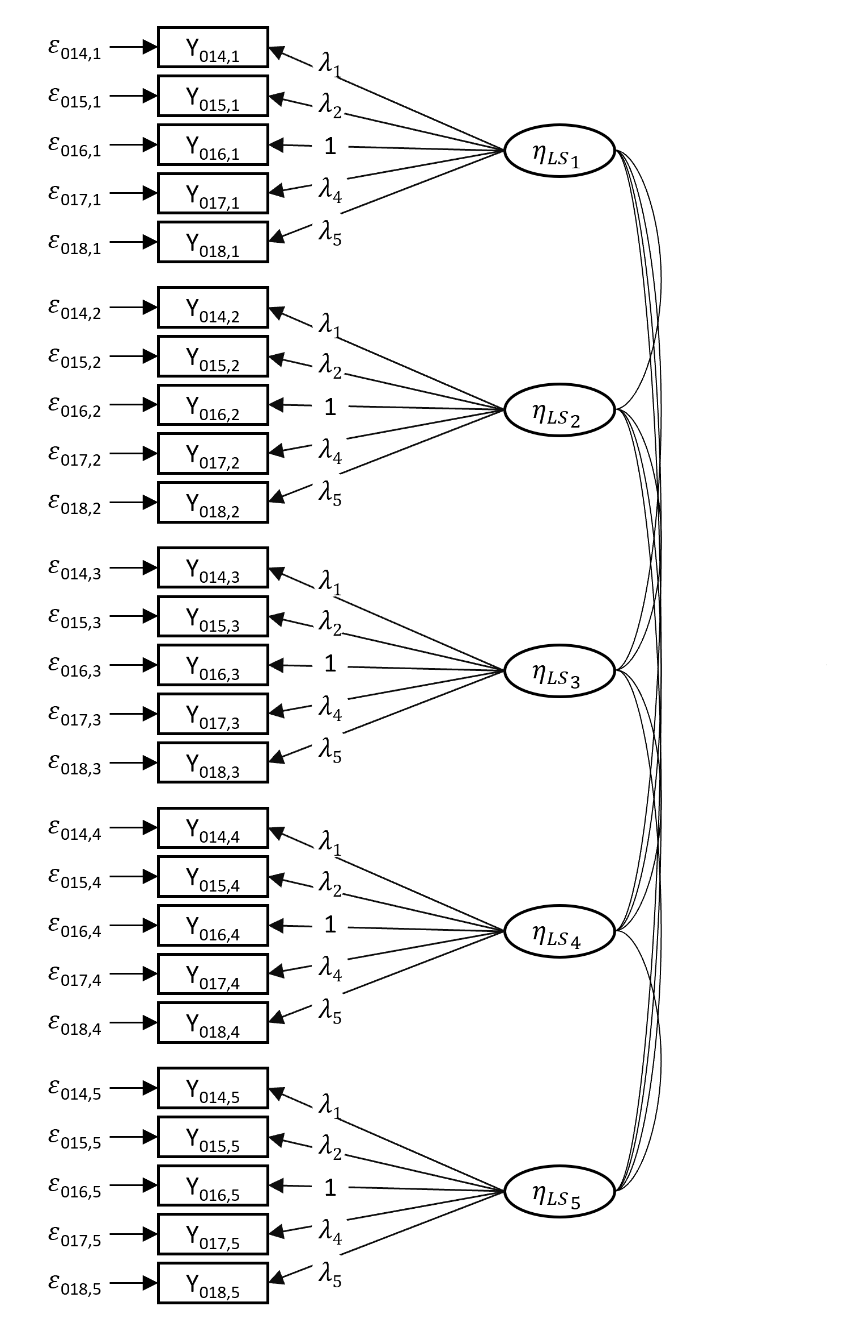


**Fig D.1. Path diagram of the multistate model of Life Satisfaction.** $\varepsilon_{it}$ measurement error, $Y_{it}$ manifest variable, ${\eta_{LS}}_{t}$ state variable for life satisfaction, $\lambda_{i}$ factor loading, $i$ number of the item, $t$ measurement occasion.


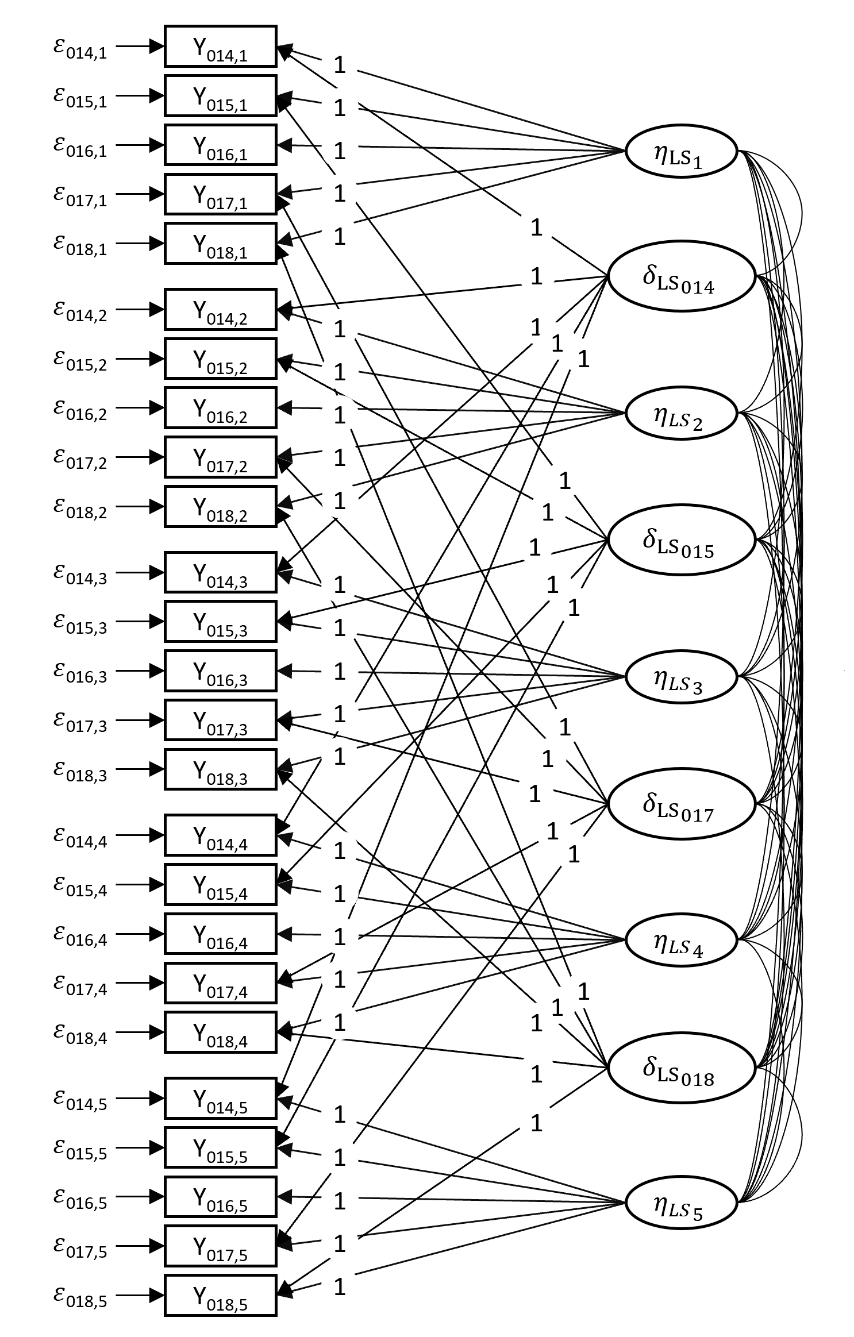


**Fig D.2. Path diagram of the multi-state model of Life Satisfaction with item effects** $\varepsilon_{it}$ measurement error, $Y_{it}$ manifest variable, ${\eta_{LS}}_{t}$state variable and ${\delta_{LS}}_{i}$item-effect variable for life satisfaction, $i$ number of the item, $t$ measurement occasion.

**Path diagram of the common model of Life Satisfaction and Positive Affect**

The path diagram in Fig D.3 represents the common model measuring five latent state variables ${\eta_{LS}}_{t}$ of life satisfaction (SWLS; Diener et al., 1985) and ${\eta_{PA}}_{t}$ of positive affect (PANAS; Watson et al., 1988). Correlations between all latent variables are considered.


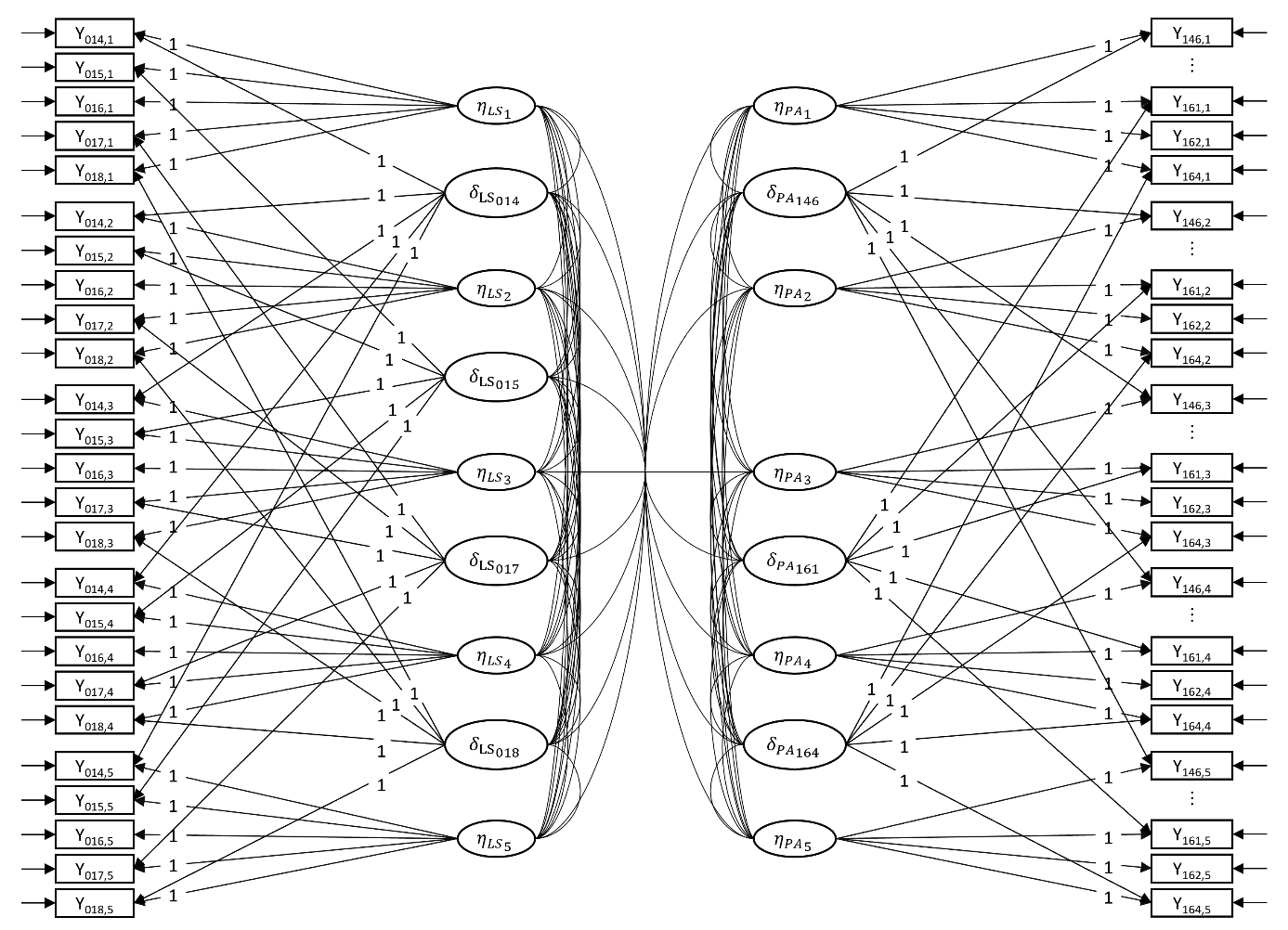


**Fig D.3. Path diagram of the common model.** $Y_{it}$ manifest variable, ${\eta_{LS}}_{t}$respectively ${\eta_{PA}}_{t}$ state variable, ${\delta_{LS}}_{i}$ respectively ${\delta_{PA}}_{i}$ item-effect variable, $LS$ life satisfaction, $PA$ positive affect, $i$ number of the item, $t$ measurement occasion. Measurement errors $\varepsilon_{it}$ are omitted in the path diagram for clarity.

**Appendix E: Model fit for unidimensional models**

For both, life satisfaction and positive affect, the application of unidimensional models at each time point was evaluated. Find the results regarding model fit in Table E1.

**Table E1. Model fit parameters for the unidimensional models for Life Satisfaction and Positive Affect at each time point.**

| Single-state model for | $\chi^{2}$ | *df* | *p-value* | RMSEA [90% CI] | | SRMR | CFI |  |
| --- | --- | --- | --- | --- | --- | --- | --- | --- |
| life satisfaction |  |  |  |  |  |  |  | |
| 2008 | 126.67 | 5 | <.001 | .10 | [.084; .113] | ***.02*** | ***.98*** | |
| 2009 | 171.26 | 5 | <.001 | .12 | [.100; 130] | ***.03*** | ***.98*** | |
| 2011 | 213.25 | 5 | <.001 | .13 | [.114; .143] | ***.03*** | ***.98*** | |
| 2013 | 141.90 | 5 | <.001 | .10 | [.090; .119] | ***.02*** | ***.98*** | |
| 2014 | 218.93 | 5 | <.001 | .13 | [.116; .145] | ***.03*** | ***.97*** | |
| positive affect |  |  |  |  |  |  |  | |
| 2008 | 1192.22 | 35 | <.001 | .11 | [.109;.120] | ***.05*** | .88 | |
| 2009 | 1193.65 | 35 | <.001 | .11 | [.109; 120] | ***.05*** | .88 | |
| 2011 | 1173.26 | 35 | <.001 | .11 | [.108; .119] | ***.05*** | .88 | |
| 2013 | 1228.48 | 35 | <.001 | .12 | [.111; .122] | ***.05*** | .89 | |
| 2014 | 1266.71 | 35 | <.001 | .12 | [.112; .124] | ***.05*** | .88 | |

*Note.* Printed in bold and italic are model fit parameters that indicate a good/acceptable model fit ($RMSEA\leq.05/.08$;$SRMR\leq.05/.10$; $CFI\geq.97/.95$; $TLI\geq.97/.95$; Schermelleh-Engel et al., 2003), CI=confidence interval.
