# Supplementary material for: Studying item-effect variables and their correlation patterns with multi-construct multi-state models
Source: PLoS One. 2023 Aug 21;18(8):e0288711. doi: 10.1371/journal.pone.0288711 (PMC10441805; doi:10.1371/journal.pone.0288711)
Supplement: S1 File — (DOCX) [file pone.0288711.s002.docx]

Electronic supplementary material

The electronic supplementary material is available under

[https://doi.org//10.17605/OSF.IO/T9735](https://doi.org/10.17605/OSF.IO/T9735)

## R Code for data preparation (.R)

## R Code for unidimensional models at each time point (.R).

## R Code for multi-state models (.R).
