# Supplementary material for: Studying item-effect variables and their correlation patterns with multi-construct multi-state models
Source: PLoS One. 2023 Aug 21;18(8):e0288711. doi: 10.1371/journal.pone.0288711 (PMC10441805; doi:10.1371/journal.pone.0288711)
Supplement: Attachment — Submitted filename: studying item effects review.pdf [file pone.0288711.s003.pdf]

The manuscript presents a multi-construct multi-state model that can be estimated from data on sets of items that measure multiple constructs that are administered at mutliple timepoints. In this way, item-effect variables can be estimated which are defined in line with Pohl (2008) as the difference between a latent-state variable that is specific to an item, and the common latent-state variable, which is the latent-state variable of a chosen reference item. The authors then apply this model to a dataset with items that measure positive affect and items that measure life satisfaction. I like that this model can help study the factors that contribute to systematic variance that is not captured in the common latent state. In general, I think this manuscript can be a nice addition to the literature on item effects. Below, I include several points and questions of clarification to help improve the presentation of the content.

General comments:

1)

About the definition of the item-effect variables: If I understand correctly, delta, the item-effect variable, is only absent in case the true-score variable of a specific item i is identical to the true-score variable of the reference item.

My question is about how the effect variables should be interpreted in the absence of systematic error. So, if I understand it correctly (but I might be mistaken), also when the congeneric model is the true model (and so there are no residual correlations) you can estimate item-effect variables because the items can differ in intercept and in vector-loading.

Is it correct that in case these true-score variables differ by a constant this would mean that only the intercepts of the items differ? (and in that case delta will not vary across people?) So, if the item effect variable does not vary across people, this can be interpreted as item a is "easier" or more easily endorsed than item b (tau_b = tau_a + intercept)?

And is it correct that in case the items have different factor loadings, this would result in an item effect variable that correlates perfectly with the true-score variable of the reference item? I am thinking, consider two items a and b and suppose that tau_a = 2*tau_b, where item a is the reference item. In that case delta = tau_a, right? or am I missing something here? If this is correct, does the correlation with the true-score variable of the reference item (the common latent-state variable) tell you something about to what extent the item-effect variable reflects differences in factor loading versus differences in systematic variance in the error/residual? And if you consider the correlation between the item-effect variable delta= tau_b-tau_a and external variables, are you then trying to explain why items a and b differ in factor loadings?

2) At several places in the manuscript sentences are gramatically incorrect or could be phrased better. Some examples: p.10 "Yet, it can obtain whether..", p.14 "For describing the result,..", p.16 "A common aspect might be content-wise", p.17 "In our application using measures.." But this is not all, so please check the whole paper.

3) The discussion section includes almost no limitations. It is mentioned that one could arrive at different conclusions when analyzing the same data with a common latent state instead of with a reference item. I would like to see a bit more discussion on what differences would be expected. For

example, if the item-effect variables are defined as residuals (instead of how they are defined in the current manuscript) is it correct that in that case there will not be any correlations between the item-effect variables and the constructs? And related to my first general comment, is it correct that in that case if items only differ in factor loadings (no systematic error) then there would not be any item-effect variable?

Do the authors see it as a problem that different ways of defining these effects would result in different conclusions? Do the authors have expectations about what conclusions would or could be different when using a common latent state instead of a reference item?

Smaller comments:

p.4: The latent state variable is introduced as τ_{it}. If I understand it correctly, since there is no subscript for the person included, this is a variable that varies over persons, rather than a constant. In that case I think it is common to use capital letter T instead of small tau to differentiate it from the true-score of a specific person, which is a constant. If the authors prefer to use tau then I would suggest to mention explicitly that τ_{it} is a variable that varies over persons, hence the missing person-subscript.

p.5 “such that the time index t can be omitted” I would add “from these parameters” since I first read it as if t was omitted completely from the model instead of that t is omitted as a subscript from the parameters since these parameters are assumed to be constant across time.

p.6 “item parameters that are constant for all persons”. I would consistently use “across” and “within”, because “for” can be ambiguous in which of the two is meant (are the item parameters constant within people over time, or across people?)

p.7 “meaning that the *differences between individual (person-specific) item effects* are stable over time” do you here mean that delta_i is stable over time? Or do you mean something else?

On p.8 it says “Error! Reference source not found. in the appendix.”

p.9 “therefore, item effects are also referred to as “systematic error”(see van Bork, 2019; van Bork et al. 2019)”. Systematic error (so when the error variable is not fully random) can indeed be modelled as an item effect variable, and the inclusion of item effect variables could be a nice way to study what kind of variables cause the systematic variance. However, the other way around, not all item effects (in the way they are defined in the current paper) are systematic variance, for the reason I mentioned in the first general comment, namely that also in the absence of systematic error you will find item-effect variables when the items differ in intercept (although in that case the item-effect variable does not vary between people) or differ in factor loading.

p.9 “concentration level” is mentioned as something that systematically affects item scores, but in contrast to the other examples of familiarity with the content and motivation, concentration level differs across occasions and in that sense is not systematic, right?

p.10 “the question of homogeneity of item effects” What is “homogeneity” in this context?

p.11 “the relevance of item effects” What is meant with “relevance” in this context? E.g., that the item-effect variable has considerable variance?

p.11, similarly “the overlap of item effects”, do you mean the correlation between item-effect variables? (these terms like homogeneity, relevance and overlap have a technical interpretation I think, but it is not clear which one).

p.13 “We consider the estimated variance of the item effect variables and the respective parameter tests against zero.” What do you mean with this sentence?

p.14 “participants were rather satisfied with their life at all five measurement occasions (η_{LS_t}= [5.45;5.58])” Do you here refer to mean of η_{LS_t}? So should this be $M_{\eta_{LS_t}}$? Similar to the SD in the same sentence? So is this the range of the means fort he latent state variable for all the different time points? (so as many means as time points?) This also applies to η_{PA_t} on p.15

p.14 The same for “(δ_{LS_i}= [-0.95;-0.24])”, should that be $M_{\delta_{LS_i}}$? So is this the range of the means for all the different items? This also applies to δ_{PA_i} on p.15

p.15 “The respective item-effect variables δ_{PA_i} indicate that, on average and in comparison” should this be “The *means of* the respective item-effect variables”? (related to previous comment)

p.15 “Thereby, we consider less that .2…” I don’t understand the “thereby”

p16 “The latent states of life satisfaction and positive affect were highly correlated within constructs” is this across items or across time? It is not completely clear what is correlated with what in this sentence. This also holds for the next sentence “In contrast, the latent state correlations..” What is correlated with what and is this across time or within a time point?

P16 “the heterogeneity of the item effects” similar to “homogeneity I am not sure what this means.

p.16 for most correlations a range is given. But fort he correlations of latent states with item effects, and then Positive affect, this range is missing.

p.17 “between the constructs, the latent states and the item effects were at most weakly correlated” does ‘between the constructs” mean that the item effects of the one construct were correlated with the latenet state of the other construct?

p.17 “Inclusion of the item-effect variables substantially improved model fit” is it the inclusion of the variables themselves? Or allowing these item-effect variables to correlate? (so that misfit due to residual correlations is solved by allowing these correlations?)

p.17 “These results imply that semantic multidimensionality is a reasonable source for the additional variation in item responses that is captured in item effects, while more global common causes are less likely” Can you explain this sentence? How do you arrive at this conclusion? and what is semantic multidimensionality?

p.18 “Specifically, positive affect defined as “attentive” was highly correlated to some item effects of the same scale” Related to my first general comment, could this indicate that the variables do not differ in systematic error but mostly differ in factor loading?
