# Supplementary material for: Studying item-effect variables and their correlation patterns with multi-construct multi-state models
Source: PLoS One. 2023 Aug 21;18(8):e0288711. doi: 10.1371/journal.pone.0288711 (PMC10441805; doi:10.1371/journal.pone.0288711)
Supplement: Attachment — Submitted filename: review revised manuscript.pdf [file pone.0288711.s006.pdf]

The authors have carefully revised the manuscript and implemented all my comments. The paper reads very well now and I was happy to see the new appendix B.

I have one more bigger point as a follow up on this appendix and its implications. In the results, variance/SD of the effect variable is interpreted: "Moreover, the participants differed considerably in the size of these item effects ($SD\delta$ = [0.39; 0.84]).", and variance is seen as an indication of the relevance of item-effect variables. But, the appendix shows that the variance of delta can also be big due to differences in factor loadings. For example, suppose that for two items i and j, $\tau_i = 5 * \tau_j$, then the standard deviation of their difference can still be considerable ($4 * \tau_j$), even though modeling an effect variable would not be needed. In this particular case you would find out because the correlation between delta and tau (or delta and eta, the reference item) would be 1. But, suppose that $\tau_i = 5 * \tau_j + x$, where x is an item specific effect of item i. Now, the correlation would no longer be 1 and so one would start to interpret the size/SD of the effect variable, but the difference in factor loading would still influence this SD a lot. That is, even if the variance of x would be small, the variance of delta would be big because of the big difference in factorloadings between item i and j. Because of this, I was wondering, do you really want the effect variable (delta) to be the difference, or would you rather want to regress the items on the reference item and have delta be the residual. (e.g., for $\tau_i = 5 * \tau_j + x$, delta would now be $4 * \tau_j + x$ but in the latter case where τ_i is regressed on τ_j , delta would only be x). Sorry, I might not have thought this completely through and it might well be that I misunderstand something or there are good reasons to prefer the first over the latter, but I hope you can explain.

Also I can understand that the reasons for choosing one over the other might be too extensive to explain in the paper (I saw some alternatives being mentioned in the discussion), but then maybe it would be good to add a note when interpreting the variance or SD of the effect variables that the way these variables are defined now means that high variance of the effect variable does not automatically mean substantial importance or relevance, in case the main explanation for te variance is differences in factor loadings (which can be investigated by analyzing the correlations?).

A few very minor things:

- Line 72 "potentially" should be "potential"

- Just to double check the interpreation of τ_{it} in equation 2: as I understand it, this is the true score variable for a specific item i and specific time t, but this variable varies over persons and situations. So, to get to a single realization of the true-score variable, one has to sample a person and a situation within time t. If this is the correct interpretation, no changes are needed :)

- Line 163-164 "modeling item effects as latent differences allows for estimating their expected values and variances" here you mean expected values and variance across situations or across people, right? Since these latent differences are stable/fixed over measurement occasions within a person x situation combination, right?

- Line 176 and line 235-236, the term ‘systematic non-state variance’ is used. In a way, couldn’t the item-specific method effects be considered state variables as well, in the sense that they vary across people and situations? (in contrast to trait variables that are stable across situations). I would think that the main contrast to the common state variable, is that these effects are item-specific as opposed to common/shared. However, I understand that “systematic item-specific state variance” (similar to item-specific traits) is not a very pretty name, so I leave it up to the authors what term they pick.

- Line 303-304 “the correlation of the item-effect variables can be investigated with regard to their size and systematic across different items and constructs.” A word seems to be missing after “systematic” or I am not sure what a “systematic” is (systematicity?). This is also the case for line 328 “provides information about the systematic of item effects”
