# Supplementary material for: Studying item-effect variables and their correlation patterns with multi-construct multi-state models
Source: PLoS One. 2023 Aug 21;18(8):e0288711. doi: 10.1371/journal.pone.0288711 (PMC10441805; doi:10.1371/journal.pone.0288711)
Supplement: Attachment — Submitted filename: Review PONE-D-22-10802.docx [file pone.0288711.s004.docx]

Review PONE-D-22-10802:

Title: Study item effects and their correlation patterns with multi-construct multi-state models

My review: I really liked the ideas presented in this article and am glad I was able to review it! The authors present a model to examine multi-construct, multi-state item-specific method effects, drawing on prior work by Koch, Geiser, Pohl, and Holtmann, among others. They mathematically define the multi-construct, multi-state model and adequately describe how to examine item/method effects within the model using a latent difference approach. Overall, I think the article is promising, the development of the method is relatively clear, and the example is appropriate. That said, I think there are areas for improvement, some general, others more specific, as noted below.

Introduction: As much as I enjoyed this paper and like what the authors have done, I feel like the introduction of the paper lacks a rationale for the development of the method, other than no one has created this method before. The introduction section needs greater justification about *why* a model, such as the one presented, is needed in contrast to both current item-specific MM models (Koch, etc.) as well as non-item-specific multi-state MM models (e.g., the multimethod latent state-trait model by Courvoisier et al., 2007). What are the benefits of the proposed model in comparison to already existing approaches? Further, I would like the authors to expand on their decision to include multiple constructs in a single model rather than examining each construct in its own model; what benefits are there to modeling multiple constructs simultaneously in this approach? Expanding on these pieces in the intro and/or discussion could help readers understand when and why to use this model compared to other similar models. Further, it may be useful to pose examples of the types of applied questions that can be answered using this model in the introduction and/or discussion section.

Specific comments:

- Eq 4. This is a specific decision to use a single item as reference within each state, following the CT-C(M-1). Why this approach and not a more generalized approach (e.g., approach following Pohl & Steyer, 2010).
- From line 100-105, there needs to be clearer separation between the error with error covariances and error with state covariances. Further, why present Eq 6 when line 104 states “all other correlations of measurement error variables are zero by definition”.
- 135-137 – No need to include this note as it’s not relevant for the current approach.
- 165: There’s an error with a reference source. Please check and fix.
- Different Sources of Multi-dimensionality section was rather difficult to read. I would encourage the authors to reorganize this section and focus on grammar and wording, especially their use of transition words (some of which are not accurate) and words such as ‘they’ and ‘their’ (which made presentation of ideas hard to follow) when describing prior work.

Results: I appreciated the clear presentation of results, but I’m curious if the authors can or have considered defining and calculating the additive variance decomposition for this modeling approach, to make the approach slightly more relevant to current LST-R approaches. Considering they assumed the items can be treated as continuous, this should be possible and may lead to informative findings that they, and others who will apply this method, can use.

- As a minor point, please report specific correlation findings for the sentence “Within positive affect…” on line 358.

Discussion: I’m curious about the specific interpretation of findings presented in the Correlations of latent states with item effects section of results. What specific item effects were found and how could they be interpreted? Although the authors describe that the inclusion of item effect variables improves model fit, what substantive meaning might the inclusion of item effects (and their correlations with state factors) have in substantive application? There is very high level discussion of this in the empirical insights section of the discussion, but I’m hoping the authors can provide specific examples from the findings reported. For example, item effect PA_148 was strongly negatively correlated with state PA variables – how might someone interpret this finding? How can these results inform current item-development practices and assessment?

Further, it’s unclear in the discussion why the development of this model is needed and necessary, similar to the introduction section. I think expanding on some of the specific findings and discussing their interpretations may help show the relevance and novelty of this method compared to other approaches.

Figures/Tables: Figure 1 does not show the model that is mathematically presented; the factor loadings need to be specified since some of them will need to be set to 1 and/or -1 (see Thielmann et al., 2017 Fig 1) for difference factor identification (see also Pohl, Steyer, & Kraus, 2008), as shown in Eq 8 (𝛿𝑖𝑡 ≔ 𝜏𝑖𝑡 − 𝜂𝑡). The mathematical presentation (and R code) of the method looks good, but figures do not reflect the mathematical presentation. Please revise the figure as well as Appendix Figures, including which loadings are fixed and which are estimated according to this approach.

General comment: Throughout the manuscript, the authors use the term “systematic error”. Pehraps, instead, use the term “systematic variance” or “systematic non-trait variance” or “systematic residual variance”, emphasizing that the systematic values being discussed are variances. As a minor semantic note, systematic error (as well as systematic error variance) is an oxymoron within LST theory, since something that is systematic is defined and interpretable, and therefore not error in the sense of CTT and LST-R.

I hope the authors find this feedback useful for improving their manuscript!

Signed, in aspiration for more accountability, transparency, and kinder peer-review practices,

Kaylee Litson, Ph.D.

Utah State University
