# Supplementary material for: Studying item-effect variables and their correlation patterns with multi-construct multi-state models
Source: PLoS One. 2023 Aug 21;18(8):e0288711. doi: 10.1371/journal.pone.0288711 (PMC10441805; doi:10.1371/journal.pone.0288711)
Supplement: Attachment — Submitted filename: Response to Reviewers.docx [file pone.0288711.s005.docx]

Dear Staff Editor Hanna Landenmark,

we greatly appreciate the effort that is spent in reviewing our manuscript PONE-D-22-10802: "Studying item-effect variables and their correlation pattern with multi-construct multi-state models". In line with the detailed and valuable suggestions of the reviewers, we have carefully revised the manuscript. We hope you share our impression, that the present version is now suitable for publication in *PLOS ONE*.

**SUMMARY OF THE EDITOR**

Thank you for submitting your manuscript to PLOS ONE. After careful consideration, we feel that it has merit but does not fully meet PLOS ONE’s publication criteria as it currently stands. Therefore, we invite you to submit a revised version of the manuscript that addresses the points raised during the review process.

Please see the comments from two reviewers below and in two attachments. Both reviewers are positive about the manuscript, and have provided specific feedback that may improve the presentation of the study.

Thank you very much for the positive feedback on the merit of our manuscript and the valuable suggestions for the revision. We carefully went through the manuscript and corrected all points raised by the reviewers. In the following, we provide detailed responses to all comments.

Journal Requirements:

When submitting your revision, we need you to address these additional requirements.

1. Please ensure that your manuscript meets PLOS ONE's style requirements, including those for file naming. The PLOS ONE style templates can be found at

<https://journals.plos.org/plosone/s/file?id=wjVg/PLOSOne_formatting_sample_main_body.pdf> and <https://journals.plos.org/plosone/s/file?id=ba62/PLOSOne_formatting_sample_title_authors_affiliations.pdf>

We carefully edited the style requirements. Specifically, we changed Headings, Figures, Tables, Supporting Information, Acknowledgements, Cover page, and References.

2. We note that you have indicated that data from this study are available upon request. PLOS only allows data to be available upon request if there are legal or ethical restrictions on sharing data publicly. For more information on unacceptable data access restrictions, please see <http://journals.plos.org/plosone/s/data-availability#loc-unacceptable-data-access-restrictions>.

In your revised cover letter, please address the following prompts:

a) If there are ethical or legal restrictions on sharing a de-identified data set, please explain them in detail (e.g., data contain potentially sensitive information, data are owned by a third-party organization, etc.) and who has imposed them (e.g., an ethics committee). Please also provide contact information for a data access committee, ethics committee, or other institutional body to which data requests may be sent.

b) If there are no restrictions, please upload the minimal anonymized data set necessary to replicate your study findings as either Supporting Information files or to a stable, public repository and provide us with the relevant URLs, DOIs, or accession numbers. For a list of acceptable repositories, please see http://journals.plos.org/plosone/s/data-availability#loc-recommended-repositories.We will update your Data Availability statement on your behalf to reflect the information you provide.

We conducted a secondary data analysis on panel data and uploading the data set is not possible. However, the data are available freely to researchers for research purposes after registration from the Dutch LISS (Longitudinal Internet Studies for the Social Sciences) panel administered by Centerdata (Tilburg University, The Netherlands). Because the data contain sensitive information, users must sign a statement to download the data from <https://www.dataarchive.lissdata.nl/>. We included this information in the manuscript in the Data availability statement.

**REVIEWERS' COMMENTS:**

**Reviewer 1:**

The manuscript presents a multi-construct multi-state model that can be estimated from data on sets of items that measure multiple constructs that are administered at multiple timepoints. In this way, item-effect variables can be estimated which are defined in line with Pohl (2008) as the difference between a latent-state variable that is specific to an item, and the common latent-state variable, which is the latent-state variable of a chosen reference item. The authors then apply this model to a dataset with items that measure positive affect and items that measure life satisfaction. I like that this mode can help study the factors that contribute to systematic variance that is not captured in the common latent state. In general, I think this manuscript can be a nice addition to the literature on item effects. Below, I include several points and questions of clarification to help improve the presentation of the content.

Thank you for highlighting that our manuscript can be a nice addition to the literature on item effects and for your very helpful suggestions on improving the manuscript

General comments:

1) About the definition of the item-effect variables: If I understand correctly, delta, the item-effect variable, is only absent in case the true-score variable of a specific item i is identical to the true-score variable of the reference item.

My question is about how the effect variables should be interpreted in the absence of systematic error. So, if I understand it correctly (but I might be mistaken), also when the congeneric model is the true model (and so there are no residual correlations) you can estimate item-effect variables because the items can differ in intercept and in vector-loading.

Is it correct that in case these true-score variables differ by a constant this would mean that only the intercepts of the items differ? (and in that case delta will not vary across people?) So, if the item effect variable does not vary across people, this can be interpreted as item a is “easier” or more easily endorsed than item b (tau_b = tau_a + intercept)?

And is it correct that in case the items have different factor loadings, this would result in an item effect variable that correlates perfectly with the true-score variable of the reference item? I am thinking, consider two items a and b and suppose that tau_a = 2*tau_b, where item a is the reference item. In that case delta = tau_a, right? or am I missing something here? If this is correct, does the correlation with the true-score variable of the reference item (the common latent-state variable) tell you something about to what extent the item-effect variable reflects differences in factor loading versus differences in systematic variance in the error/residual? And if you consider the correlation between the item-effect variable delta= tau_b-tau_a and external variables, are you then trying to explain why items a and b differ in factor loadings?

Thank you for carefully considering the definition and interpretation of the item-effect variables and their correlations. The suggestion of the reviewer on the impact of different factor loadings was very helpful for describing the interpretation of the correlations. As the reviewer outlines, we now also consider the special case when a congeneric model holds, for linking the parameters of the item-effect variables to the intercept and loading parameters in congeneric models. This is very helpful for better understanding the more general case when a congeneric model does not hold and systematic non-state variance occurs in item responses due to other person characteristics and their interaction with the items.

Specifically, in the revised manuscript, we describe the different interpretations of the correlations with item-effect variables in more detail and we delineate different scenarios for the correlations in Appendix B.

**Appendix B: Different scenarios for zero or perfect correlations with item-effect variables**

For measuring one construct at different measurement occasions with a multi-item scale, we define latent state variables $\eta_{t}:=\tau_{1t}$ (i.e., the latent ability at a specific time point $t$, measured with the state variable of the first item that is chosen as the reference item) and latent item-effect variables $\delta_{it}:={\tau_{it}-\tau}_{1t}$ (i.e., the latent person-specific difference of using another item $i$ instead of the reference item for measuring the latent construct).

Of interest are the correlations between the latent state variables and item-effect variables, that are, $Cor(\eta_{t},\delta_{it})$for different time points $t$ and items $i$, and among the item-effect variables of different items $i$ and $k,$that are $Cor(\delta_{it},\delta_{kt})$. For describing the meaning of the correlations, we first consider special cases without person-specific differences between the items, but constant differences across all persons. Specifically, either an essentially equivalent measurement model holds (i.e., $\tau_{it}={\nu_{it}+\tau}_{1t}$), where items differ in an intercept $\nu_{it}$ , or a congeneric model holds (i.e., $\tau_{it}={\nu_{it}+\lambda_{it}\tau}_{1t}$), where items differ in an intercept $\nu_{it}$ and factor loading $\lambda_{it}$. In these cases, the parameters of the item-effect variables can directly be linked to the intercepts and factor loadings.

Under the assumption of an essentially equivalent measurement model, it can be shown that,

$${E(\delta}_{it})=E\left( {\tau_{it}-\tau}_{1t} \right)=E\left( {\nu_{it}+\tau}_{1t}-\tau_{1t} \right)=\nu_{it},$$

$${Var(\delta}_{it})=Var\left( \tau_{it}-\tau_{1t} \right)=Var\left( \nu_{it}+\tau_{1t}-\tau_{1t} \right)=0,$$

the expected value of the item-effect variables corresponds to the intercept and their variance is zero. Due to a zero variance, all correlations with the item-effect variables are zero, too. Thus, in this case, no systematic variance is prevalent and can be explained.

Instead, when a congeneric model holds, then,

$${Var(\delta}_{it})=Var(\tau_{it}-\tau_{1t})=Var\left( \nu_{it}+\lambda_{it}\tau_{1t}-\tau_{1t} \right)=\left( \lambda_{it}-1 \right)^{2}Var\left( \tau_{1t} \right),$$

systematic variation is present in relation to the loading parameter. Yet, this variation can perfectly be explained by the latent state, whereas the covariance is,

$$Cov\left( \eta_{t},\delta_{it} \right)=Cov\left( \tau_{1t},\nu_{it}+\lambda_{it}\tau_{1t}-\tau_{1t} \right)=Cov\left( \tau_{1t},\tau_{1t}(\lambda_{it}-1) \right) =\left( \lambda_{it}-1 \right)Var\left( \tau_{1t} \right),$$

that can be inserted in the formula for the correlation,

$$Cor\left( \eta_{t},\delta_{it} \right)=\frac{Cov\left( \eta_{t},\delta_{it} \right)}{\sqrt{Var(\eta_{t})}\sqrt{Var(\delta_{it})}}=\frac{\left( \lambda_{it}-1 \right)Var\left( \tau_{1t} \right)}{\sqrt{Var\left( \tau_{1t} \right)}\sqrt{\left( \lambda_{it}-1 \right)^{2}Var\left( \tau_{1t} \right)}}=\pm1.$$

Note, in cases where the factor loading is negative, the correlation would be negative, too. Similarly, the correlations among item-effect variables of different items $i$ and $k$, for which a congeneric model holds can be derived, with the covariance,

$$Cov\left( \delta_{it},\delta_{kt} \right)=Cov\left( \nu_{it}+\lambda_{it}\tau_{1t}-\tau_{1t},\nu_{kt}+\lambda_{kt}\tau_{1t}-\tau_{1t} \right)=\left( \lambda_{it}-1 \right)\left( \lambda_{kt}-1 \right)Var\left( \tau_{1t} \right),$$

that can be inserted in the formula for the correlation,

$$Cor\left( \delta_{it},\delta_{kt} \right)=\frac{Cov\left( \delta_{it},\delta_{kt} \right)}{\sqrt{Var(\delta_{it})}\sqrt{Var(\delta_{kt})}}=\frac{\left( \lambda_{it}-1 \right)\left( \lambda_{kt}-1 \right)Var\left( \tau_{1t} \right)}{\sqrt{\left( \lambda_{it}-1 \right)^{2}Var\left( \tau_{1t} \right)}\sqrt{\left( \lambda_{kt}-1 \right)^{2}Var\left( \tau_{1t} \right)}}=\pm1.$$

Accordingly, in this case, there is one common source for the systematic variation between items, that is the latent state itself, due to different loadings. If one loading is negative, the correlation would be negative, too.

In the described special cases, it would not be necessary to model item-effect variables. Instead, when a congeneric model does not hold, then items differ not only in constant parameters and the item effect variables $\delta_{it}:=\tau_{it}-\tau_{1t}$ cannot be simplified in form of a linear transformation of the latent state variable. This is the more general case, in which various causes for the item-effect variables can be present. The correlations of item-effect variables with the states $Cor\left( \eta_{t},\delta_{it} \right)$ indicate whether the states are a possible explanation for the systematic non-state variance, due to differences in factor loadings or common explanatory variables. Also, the correlations among item-effect variables of different items $Cor\left( \delta_{it},\delta_{kt} \right)$ indicate whether common or comparable causes (e.g., the latent state variables as well as other person characteristics) are plausible. An example of a common cause for item-effect variables of two different items would be a wording effect (i.e., the reference item is a positive statement, but the items $i$and $k$ are negatively formulated). If such a wording effect is the only cause for differences between items, it can be modeled as a common method variable $W=\tau_{it}-\tau_{1t}=\tau_{kt}-\tau_{1t}$, that is similar for both items (or a linear transformation in a more general case). In this case, the correlation of the latent state variable with the item-effect variables depends on the correlation with $W$,

$$Cor\left( \eta_{t},\delta_{it} \right)=\frac{Cov\left( \eta_{t},\delta_{it} \right)}{\sqrt{Var(\eta_{t})}\sqrt{Var(\delta_{it})}}=\frac{Cov\left( \eta_{t},W \right)}{\sqrt{Var\left( \tau_{1t} \right)}\sqrt{Var\left( W \right)}}$$

In the most extreme case, the correlation of the latent state variables with the item-effect variables is zero, which is often assumed in residual definitions of method effects. Meaning the method effect and the latent state are distinct variance components that systematically affect the item responses. Instead, the correlation of the item-effect variables is still perfect in this case,

$$Cor\left( \delta_{it},\delta_{kt} \right)=\frac{Cov\left( \delta_{it},\delta_{kt} \right)}{\sqrt{Var(\delta_{it})}\sqrt{Var(\delta_{kt})}}=\frac{Var(W)}{\sqrt{Var(W)}\sqrt{Var(W)}}=\pm1,$$

Note, if the item-effect variable of one item is a negative transformation of $W$, the correlation would be negative, too. The correlation among item-effect variables reduces in its absolute value, and the more distinct the cause for different item-effect variables are. It can be zero in the most extreme case of completely distinct causes for item-effect variables of different items.

2) At several places in the manuscript sentences are gramatically incorrect or could be phrased better. Some examples: p.10 “Yet, it can obtain whether..”, p.14 “For describing the result,..”, p.16 “A common aspect might be content-wise”, p.17 “In our application using measures..”But this is not all, so please check the whole paper.

Thank you for pointing out that some sentences need to be rephrased. We carefully edited the whole manuscript.

3) The discussion section includes almost no limitations. It is mentioned that one could arrive at different conclusions when analyzing the same data with a common latent state instead of with a reference item. I would like to see a bit more discussion on what differences would be expected. For example, if the item-effect variables are defined as residuals (instead of how they are defined in the current manuscript) is it correct that in that case there will not be any correlations between the item-effect variables and the constructs? And related to my first general comment, is it correct that in that case if items only differ in factor loadings (no systematic error) then there would not be any item-effect variable?

Do the authors see it as a problem that different ways of defining these effects would result in different conclusions? Do the authors have expectations about what conclusions would or could be different when using a common latent state instead of a reference item?

Yes, the identification scheme for specifying the latent variables determines their interpretation and correlation pattern. This holds for our approach, as well as for congeneric models without item effects or other MTMM approaches. We included a discussion on the strengths and limitations of our approach and more carefully draw on the impact of alternative modeling approaches for the derived conclusions. Specifically, we describe that:

For using the reference coding scheme, the selection of the reference item should be plausible for a specific instrument; we relied on substantive theory and considered items that captured the corresponding construct most directly, although this was less clear for the positive affect scale. Using another reference item will not affect model fit, but the interpretation of the latent variables. Similarly, an alternative parametrization using an effect coding scheme can be applied for specifying average states across all items [see 33, 14,]. The item-effect variables will always represent the difference to the latent states, but the interpretation of the item-effect variables depends on the meaning of the latent states. Thus, analyzing the same data with another identification scheme will enable different interpretations in subsequent analyses. Our approach of using a global reference item might support the occurrence of item-effect variables that capture more distinct aspects of other items in the sense of semantic multi-dimensionality. Instead using a more specific reference item may result in more similarities between the item-effect variables (i.e., they share variation from a specific item). The specification of common latent states would entangle the content of different items, thus, comparisons between specific items would be limited.

Smaller comments:

p.4: The latent state variable is introduced as 𝜏_𝑖𝑡_. If I understand it correctly, since there is no subscript for the person included, this is a variable that varies over persons, rather than a constant. In that case I think it is common to use capital letter T instead of small tau to differentiate it from the true-score of a specific person, which is a constant. If the authors prefer to use tau then I would suggest to mention explicitly that 𝜏_𝑖𝑡_ is a variable that varies over persons, hence the missing person-subscript.

Thank you for highlighting that the notation requires a more detailed description. The reviewer is right that latent state variables$\eta_{it}:=\tau_{1t}$, as well as the latent item-effect variables $\delta_{it}$, the manifest variables $Y_{it}$, and the measurement error variables $\varepsilon_{it}$ vary over persons. For denoting the variables, we use the notation of the LST theory, like in Steyer et al. (2015), Thielemann et al. (2017), or Pohl & Steyer (2010). According to this notation, no person-subscript is included, but the specification as random variables describes the variation across persons. As the reviewer points out, different traditions for denoting the variables are prevalent in the literature (e.g., Eid, 2000 uses the capital letter T for denoting a trait). Thus, to make the point clearer that all variables vary over persons, we explicitly introduce them with the statement “All variables can take on different values for different persons.”. In addition, we now describe our specification of item effects as item-effect variables, to be more congruent with the concept of variables.

p.5 “such that the time index t can be omitted” I would add “from these parameters” since I first read it as if t was omitted completely from the model instead of that t is omitted as a subscript from the parameters since these parameters are assumed to be constant across time.

We included this clarification in the manuscript. Thank you.

p.6 “item parameters that are constant for all persons”. I would consistently use “across” and “within”, because “for” can be ambiguous in which of the two is meant (are the item parameters constant within people over time, or across people?)

We also included this clarification throughout the manuscript. Thank you.

p.7 “meaning that the *differences between individual (person-specific) item effects* are stable over time” do you here mean that delta_i is stable over time? Or do you mean something else?

Yes, $\delta_{i}$ is constant within each person across time. We clarified this point in the manuscript.

On p.8 it says “Error! Reference source not found. in the appendix.”

We are sorry for the inconvenience. In the revised manuscript, we corrected the error message and now refer the reader to Table A1 in the Appendix.

p.9 “therefore, item effects are also referred to as “systematic error” (see van Bork,2019; van Bork et al. 2019)”. Systematic error (so when the error variable is not fully random) can indeed be modelled as an item effect variable, and the inclusion of item effect variables could be a nice way to study what kind of variables cause the systematic variance. However, the other way around, not all item effects (in the way they are defined in the current paper) are systematic variance, for the reason I mentioned in the first general comment, namely that also in the absence of systematic error you will find item-effect variables when the items differ in intercept (although in that case the item-effect variable does not vary between people) or differ in factor loading.

Indeed, in the special case when a congeneric model holds, the item-effect variables would represent just the differences in intercepts and factor loadings. Thank you for pointing this out. We clarified the respective paragraph in line with our response to the first comment of the reviewer.

p.9 “concentration level” is mentioned as something that systematically affects item scores, but in contrast to the other examples of familiarity with the content and motivation, concentration level differs across occasions and in that sense is not systematic, right?

Right, the example can be misleading. We intended that concentration level can differ between persons and can systematically affect the item responses. For example, a less concentrated person may miss a negatively formulated item. However, as the reviewer notes, the concentration level is often not stable over time. As such, we deleted this example to avoid confusion.

p.10 “the question of homogeneity of item effects” What is “homogeneity” in this context?

Thank you for pointing out that the terms homogeneity and heterogeneity are misleading. In the revised manuscript, we now specify that we investigate the size and systematic of the correlations of item-effect variables across different items and constructs.

p.11 “the relevance of item effects” What is meant with “relevance” in this context? E.g., that the item-effect variable has considerable variance?

We tested the relevance of item-effect variables first by comparing the fit of a congeneric model without item-effect variables to a model with item-effect variables. Then, we investigated the size of the variance of each item-effect variable. In the revised manuscript, we point out that both criteria are important for investigating the relevance of item effects, in order to study if a congeneric model would be sufficient and if some item-effect variables are neglectable.

p.11, similarly “the overlap of item effects”, do you mean the correlation between item-effect variables? (these terms like homogeneity, relevance and overlap have a technical interpretation I think, but it is not clear which one).

Thank you for clarifying that we have to be more consistent with these terms throughout the manuscript. In the revised manuscript we only use the terms relevance (i.e., for describing whether item-effect variables are neglectable in an application or not) and correlation (i.e., for describing similarities between different variables and to if different items share systematic effects).

p.13 “We consider the estimated variance of the item effect variables and the respective parameter tests against zero.” What do you mean with this sentence?

We edited the sentence for clarification: To identify whether specific item-effect variables are negligible, we used the test statistic of the estimated variance divided by its standard error for testing the parameter against zero.

p.14 “participants were rather satisfied with their life at all five measurement occasions (𝜂_𝐿𝑆𝑡_= [5.45;5.58])” Do you here refer to mean of 𝜂_𝐿𝑆𝑡_? So should this be 𝑀_𝜂𝐿𝑆𝑡_? Similar to the SD in the same sentence? So is this the range of the means for the latent state variable for all the different time points? (so as many means as time points?) This also applies to 𝜂_𝑃𝐴𝑡_ on p.15

p.14 The same for “(𝛿_𝐿𝑆𝑖_= [-0.95;-0.24])”, should that be 𝑀_𝛿𝐿𝑆𝑖_? So is this the range of the means for all the different items? This also applies to 𝛿_𝑃𝐴𝑖_ on p.15

Yes, thank you. In Table 3, we included the$M$ for referring to the means of the respective variables and included a statement for the range: In the following, the numbers in brackets describe the range of the estimated parameters across measurement occasions $t$ for the state variables (i.e., ${\eta_{LS}}_{t}$, ${\eta_{PA}}_{t})$and across items $i$ for the item-effect variables (i.e., ${\delta_{LS}}_{i}$ or ${\delta_{PA}}_{i})$.

p.15 “The respective item-effect variables 𝛿_𝑃𝐴𝑖_ indicate that, on average and in comparison” should this be “The means of the respective item-effect variables”? (related to previous comment)

Yes, thank you, we now refer to the mean.

p.15 “Thereby, we consider less that .2...” I don’t understand the “thereby”

Thank you, we deleted the word.

p16 “The latent states of life satisfaction and positive affect were highly correlated within constructs” is this across items or across time? It is not completely clear what is correlated with what in this sentence. This also holds for the next sentence “In contrast, the latent state correlations.” What is correlated with what and is this across time or within a time point?

Indeed, we need to be more precise in reporting the correlations. We carefully edited this part and the following parts with clear notations:

The latent state variables of life satisfaction and positive affect were highly correlated within constructs across different time points $t$ and $s$ (for life satisfaction:$Cor\left( \eta_{LS_{t}},\eta_{LS_{s}} \right)=\left[ .54;.79 \right]$; for positive affect: $Cor\left( \eta_{PA_{t}},\eta_{PA_{s}} \right)=\left[ .65;.74 \right]$). In contrast, the latent state correlations between constructs, within and across time points were negligible to small$(Cor\left( \eta_{PA_{t}},\eta_{LS_{s}} \right)=\left[ .08;.25 \right])$. Thus, both constructs, as defined with the reference item, were rather stable over the five measurement occasions, but hardly explained each other.

P16 “the heterogeneity of the item effects” similar to “homogeneity I am not sure what this means.

Right, we now directly refer to the systematic of the correlations involving item-effect variables.

p.16 for most correlations a range is given. But for the correlations of latent states with item effects, and then Positive affect, this range is missing.

Thank you for pointing this out, we added the respective range to the manuscript.

p.17 “between the constructs, the latent states and the item effects were at most weakly correlated” does ‘between the constructs” mean that the item effects of the one construct were correlated with the latent state of the other construct?

Yes, we corrected the sentence: Between the constructs, the variables were at most weakly correlated (i.e., $Cor({\eta_{PA_{t}},\delta}_{LS_{i}})=\left[ -.07;.02 \right]$ and $Cor\left( {\eta_{LS_{t}},\delta}_{PA_{i}} \right)=\left[ -.25;.25 \right]$).

p.17 “Inclusion of the item-effect variables substantially improved model fit” is it the inclusion of the variables themselves? Or allowing these item-effect variables to correlate? (so that misfit due to residual correlations is solved by allowing these correlations?)

We refer to the complete model with item-effect variables and their correlations. Accordingly, we rephrased the sentence.

p.17 “These results imply that semantic multidimensionality is a reasonable source for the additional variation in item responses that is captured in item effects, while more global common causes are less likely” Can you explain this sentence? How do you arrive at this conclusion? and what is semantic multidimensionality?

In line with the other comments of the reviewer, we carefully edited our presentation of the results. Here we also draw on the specific correlation pattern and outline:

For the specified item-effect variables in the SWLS, we obtained no substantial correlation to the variables of the positive affect scale (i.e., states or item-effect variables between constructs) or to the general measure of life satisfaction (i.e., states within the construct). Only for some item-effect variables within the construct, we found similarities in relation to the specific item content. These results support the interpretation of item-effect variables in the sense of semantic multidimensionality (i.e., the item-effect variables reflect unique domain content for the focal construct in relation to the item content). Yet, other explanations cannot be ruled out. The correlation pattern is slightly different for the specified item-effect variables in the positive affect scale. Like for life satisfaction, all substantial correlations were rather construct-specific and more global common causes of item-effect variables are less likely (i.e., no substantial correlations to states or item-effect variables between constructs). However, for some items, the item-effect variables were substantially correlated to the states within the construct. In addition, we found some similarities among the item-effect variables within the construct in relation to the item content. As such, semantic multidimensionality may be present for some items, but possibly also differences in relation to the states (i.e., different factor loadings, or more substantive explanations for item-effect variables due to ‘attentive’ positive affect as well as related constructs). Again, further explanations for item-effect variables cannot be ruled out.

p.18 “Specifically, positive affect defined as “attentive” was highly correlated to some item effects of the same scale” Related to my first general comment, could this indicate that the variables do not differ in systematic error but mostly differ in factor loading?

Yes, we added the explanation of different factor loadings for item-effect variables throughout the manuscript. It is one possible explanation for substantial correlations of item-effect variables and states within the construct. However, more substantive explanations are possible, too. For instance, those item-effect variables occur due to being more or less attentive or due to common causes of item-effect variables and attentive positive affect. We more carefully point out different explanations in the revised manuscript.

**Reviewer 2:**

My review: I really liked the ideas presented in this article and am glad I was able to review it! The authors present a model to examine multi-construct, multi-state item-specific method effects, drawing on prior work by Koch, Geiser, Pohl, and Holtmann, among others. They mathematically define the multi-construct, multi-state model and adequately describe how to examine item/method effects within the model using a latent difference approach. Overall, I think the article is promising, the development of the method is relatively clear, and the example is appropriate. That said, I think there are areas for improvement, some general, others more specific, as noted below.

Thank you for valuing the general idea of our manuscript and the mathematical definition as well as the application of the model. Your comments are very helpful for improving the manuscript, as we describe below.

Introduction: As much as I enjoyed this paper and like what the authors have done, I feel like the introduction of the paper lacks a rationale for the development of the method, other than no one has created this method before. The introduction section needs greater justification about *why* a model, such as the one presented, is needed in contrast to both current item-specific MM models (Koch, etc.) as well as non-item-specific multi-state MM models (e.g., the multimethod latent state-trait model by Courvoisier et al., 2007). What are the benefits of the proposed model in comparison to already existing approaches?

Thank you for this clear suggestion. We extended the introduction and carefully consider other approaches. As such, we outline why the presented approach of modeling item-specific method effects as latent differences is well-suited with regard to our research focus – that is gaining a better understanding of the measurement process by investigating the correlation pattern in multi-construct contexts.

Specifically, we describe that non-item-specific MTMM models (e.g., Courvoisier, 2006; Courvoisier et al., 2008) assume a common method effect for multiple items. As such, they have a clear interpretation of the specific method effect, but can be too strict. Modeling differences on the item-level is more detailed and can be implemented in form of item-specific traits (e.g., Eid & Kutscher, 2014; Joshanloo, 2022; López-Benitez et al., 2019; Scarpato et al., 2021) or item-specific method effects (e.g., Cogo-Moreira et al., 2021; Geiser et al., 2019; Holtmann et al., 2020; Thielemann et al., 2017). Method effects separate the item-specificity from effects that are common for all items and are well-suited for further investigating the item-specific effects themselves. The approach of modeling item effects as regression residuals is typically used for investigating additive variance components, that is, for describing the method-specificity of a measure. Therefore, a common assumption is that the residuals and states are uncorrelated. This assumption is not necessary and without restrictions on the correlation structure, the residual approach is closely aligned with our approach of modeling latent differences (see e.g., Geiser & Lockhart, 2012). When modeling method effects as latent differences, typically all correlations are estimated and we show how they can be used for further investigating the item effects.

Further, I would like the authors to expand on their decision to include multiple constructs in a single model rather than examining each construct in its own model; what benefits are there to modeling multiple constructs simultaneously in this approach? Expanding on these pieces in the intro and/or discussion could help readers understand when and why to use this model compared to other similar models. Further, it may be useful to pose examples of the types of applied questions that can be answered using this model in the introduction and/or discussion section.

Thank you for pointing out that the benefit of our work should be clearer. We included more details in the introduction and more carefully draw on the benefits in the discussion.

The advantages of our approach are:

- All latent variables are specified based on LST theory. Item-effect variables are modeled as stable differences between an item and the reference item and we only use necessary model assumptions that specify measurement invariance across time points. Thus, all latent correlations can be further investigated.
- We investigated the correlation pattern for similarities and differences between item-effect variables within and between constructs. This can help to identify, whether common causes for the systematic non-state variation are plausible. Especially, if this is the case for different constructs the nature of item effects is more global and less related to the measurement of a specific construct.

Based on this, our approach can be used for different research questions:

- Psychometric research on scale construction can study the dimensionality in multi-construct scales and can identify, whether more strict measurement assumptions are plausible (e.g., for modeling a common method effect for specific items).
- Methodological research questions can evaluate the occurrence of item-specific method effects under different conditions (e.g., in relation to the item format, different response scales, or item characteristics) and can investigate whether item effects generalize across different constructs.
- Substantive research (e.g., on the contribution of item-effect variables for predictive validity) can gain a better understanding of similarities and possible explanations of item-effect variables. This can help in judging whether the item-effect variables represent primary semantic or method variance in relation to the item content, and respectively may be more or less useful for substantive analysis.

Specific comments:

- Eq 4. This is a specific decision to use a single item as reference within each state, following the CT-C(M-1). Why this approach and not a more generalized approach (e.g., approach following Pohl & Steyer, 2010).

Indeed, both approaches (i.e., defining a common or reference state) are possible and equivalent with regard to model fit (see e.g., Pohl & Steyer, 2010). We chose the specification with a reference coding scheme because the latent states as well as the item-effect variables are directly related to the specific item content. With an effect coding scheme, the latent states would represent the mean of all items and the item-effect variables the difference to this mean. This is also plausible but would indicate another meaning of the states and item-effect variables. We now draw on this alternative approach in the discussion.

- From line 100-105, there needs to be clearer separation between the error with error covariances and error with state covariances. Further, why present Eq 6 when line 104 states “all other correlations of measurement error variables are zero by definition”.

Yes, this part requires some more explanations, because assumptions for the error variables are more complex in the revised LST theory in comparison to its former presentation. The idea is, that the person variable $U_{t}$ depends on the time point $t$ and includes previous experiences of the persons. Assumptions on error correlations as well as on error and state correlations depend on the time point, too. Depending on the time point, some of the correlations are zero by definition, but others need to be restricted to zero as additional model assumptions. We have made the separation clearer and described the specific meaning of Eq. 6 and 7 in the revised manuscript.

By definition, in the revised LST theory [17] all correlations between measurement error variables of different time points, as well as between measurement error variables and states of the same or earlier time points are zero. Furthermore, the multi-state model requires that all measurement error variables of different items within a time point $t$ are uncorrelated and measurement error variables are uncorrelated with future states:

|  | $cov\left( \varepsilon_{it},\varepsilon_{jt} \right)=0, \forall i\neq j$ with $i,j=1,\ldots,m,$ $\forall t=1,\ldots,n,$ | (6) |
| --- | --- | --- |
|  | $cov\left( \varepsilon_{it},\tau_{is} \right)=0, \forall i=1,\ldots,m$ at $s>t$. | (7) |
| - 135-137 – No need to include this note as it’s not relevant for the current approach. | | |

When defining a multi-state model with item-effect variables, then all factor loadings are set to 1 and all intercepts to 0, such that the item-effect variables represent the differences between items (i.e., $\tau_{it}=\eta_{t}+\delta_{it}$). We rephrased the sentence and included the note on the factor loadings and intercepts, as it is relevant to the current approach.

- 165: There’s an error with a reference source. Please check and fix.

We are sorry, for the inconvenience. In the revised manuscript, we corrected the error and now refer the reader to Table A1 in the Appendix.

- Different Sources of Multi-dimensionality section was rather difficult to read. I would encourage the authors to reorganize this section and focus on grammar and wording, especially their use of transition words (some of which are not accurate) and words such as ‘they’ and ‘their’ (which made presentation of ideas hard to follow) when describing prior work.

Thank you very much for this clear suggestion. We carefully revised this section – also in line with the comments of the other reviewer on the meaning of semantic multidimensionality.

Results: I appreciated the clear presentation of results, but I’m curious if the authors can or have considered defining and calculating the additive variance decomposition for this modeling approach, to make the approach slightly more relevant to current LST-R approaches. Considering they assumed the items can be treated as continuous, this should be possible and may lead to informative findings that they, and others who will apply this method, can use.

Indeed, investigating additive variance decomposition for the latent states and item-effect variables would be interesting. However, as latent state variables and item-effect variables are correlated in our approach, this decomposition is not possible, so far. We draw on this difference to modeling method effects as residuals in the introduction, as well as in the discussion. In order to study the relevance of item-effect variables with our approach, we consider the significance and size of their variance.

- As a minor point, please report specific correlation findings for the sentence “Within positive affect…” on line 358.

Thank you for pointing this out, we added the specific correlation findings.

Discussion: I’m curious about the specific interpretation of findings presented in the Correlations of latent states with item effects section of results. What specific item effects were found and how could they be interpreted? Although the authors describe that the inclusion of item effect variables improves model fit, what substantive meaning might the inclusion of item effects (and their correlations with state factors) have in substantive application? There is very high level discussion of this in the empirical insights section of the discussion, but I’m hoping the authors can provide specific examples from the findings reported. For example, item effect PA_148 was strongly negatively correlated with state PA variables – how might someone interpret this finding? How can these results inform current item-development practices and assessment?

In the revised manuscript, we more carefully describe the interpretation of the correlations in our application of the multi-state multi-construct model with item-specific effects. In line with the comments of the other reviewer, we carefully considered different factor loadings as one explanation for the strong correlation between the latent states and some item-effect variables within positive affect. In addition, we described the results with more relation to the item content. In the discussion, we still consider a higher level for presenting the results as they are very specific for the considered scales in our application.

Further, it’s unclear in the discussion why the development of this model is needed and necessary, similar to the introduction section. I think expanding on some of the specific findings and discussing their interpretations may help show the relevance and novelty of this method compared to other approaches.

Yes, in line with the introduction and theory section we also edited the discussion. Specifically, we describe the benefit of the presented model in the new section strengths and limitations. Furthermore, we more carefully consider the differences to alternative modeling approaches in the respective section of the discussion.

Figures/Tables: Figure 1 does not show the model that is mathematically presented; the factor loadings need to be specified since some of them will need to be set to 1 and/or -1 (see Thielmann et al., 2017 Fig 1) for difference factor identification (see also Pohl, Steyer, & Kraus, 2008), as shown in Eq 8 (𝛿𝑖𝑡 ≔ 𝜏𝑖𝑡 − 𝜂𝑡). The mathematical presentation (and R code) of the method looks good, but figures do not reflect the mathematical presentation. Please revise the figure as well as Appendix Figures, including which loadings are fixed and which are estimated according to this approach.

The figures, so far, just represented the configural models. We included factor loadings in all figures.

General comment: Throughout the manuscript, the authors use the term “systematic error”. Perhaps, instead, use the term “systematic variance” or “systematic non-trait variance” or “systematic residual variance”, emphasizing that the systematic values being discussed are variances. As a minor semantic note, systematic error (as well as systematic error variance) is an oxymoron within LST theory, since something that is systematic is defined and interpretable, and therefore not error in the sense of CTT and LST-R.

We agree with the reviewer that the suggested terms are better suited for describing the variance of item-effect variables. We used the term “systematic error” in line with the work of

van Bork, R. (2019). Interpreting psychometric models [Doctoral thesis, University of Amsterdam]. Thesis Commons. <https://doi.org/10.31237/osf.io/x6a7s>

van Bork, R., Rhemtulla, M., Sijtsma, K., & Borsboom, D. (2022). A causal theory of error scores. *Psychological Methods*. Advance online publication. https://doi.org/10.1037/met0000521

who specified systematic error as the variance of stable person characteristics in item responses next to the latent attribute of interest. Accordingly, we use the term “systematic non-state variance” in the revised manuscript and describe the relation to the work of van Bork and colleagues.

I hope the authors find this feedback useful for improving their manuscript!

Signed, in aspiration for more accountability, transparency, and kinder peer-review practices,

Kaylee Litson, Ph.D.

Utah State University

Thank you very much Dr. Litson for the very helpful feedback.
