# Supplementary material for: Studying item-effect variables and their correlation patterns with multi-construct multi-state models
Source: PLoS One. 2023 Aug 21;18(8):e0288711. doi: 10.1371/journal.pone.0288711 (PMC10441805; doi:10.1371/journal.pone.0288711)
Supplement: Attachment — Submitted filename: Response to Reviewers.docx [file pone.0288711.s007.docx]

Dear Guest Editor Kaylee Litson,

Thank you very much for guest editing our manuscript PONE-D-22-10802R1: "Studying item-effect variables and their correlation pattern with multi-construct multi-state models". We greatly appreciate the careful second review round. In the revision, we carefully considered all points and hope you share our impression that the present version meets *PLOS ONE*’s publication criteria.

**SUMMARY OF THE EDITOR**

I was asked to guest edit this manuscript after my initial review as Reviewer 2. The paper has addressed both mine and Reviewer 1’s comments appropriately, and I enjoyed reading the revised manuscript. Like Reviewer 1, I appreciate the new addition of Appendix B and its overview of the modeling approach. Further, the expanded, edited introduction and discussion sections highlight the relevance of this new approach, and the additional clarity in results makes a strong contribution to this piece. Only minor revisions are requested prior to publication, and the necessary revisions are noted in the comments below.

Thank you very much for the positive feedback on our former edits and the clear suggestions for the additional revision. We carefully went through the manuscript and corrected all points raised by the editor and reviewer 1. In the following, we provide detailed responses to all comments.

Reviewer 1 describes an important concern about the mathematical configuration of the single construct models without item-specific factors that are presented in Appendix B (please see their review for an entire overview). They note, and I also see, that the variance of delta is intertwined with differences in factor loadings within the single construct models without item specific effects. They then make a suggestion about regressing the items on the reference item and changing delta to be the residual. This could be an important avenue for future work, yet in my own reading of this paper, this factor loading issue they raise is only relevant in the single construct models without item specific effects; in the manuscript, you seem to interpret the single construct models with item specific effects, as well as the multi-construct models with item specific effects, which have all factor loadings constrained to 1. Thus, this issue about factor loadings impacting correlation coefficients does not need to be addressed in the revision of the current manuscript, unless you feel inclined to address it.

We carefully considered the suggestions of Reviewer 1, whereas we describe the impact of different factor loadings for the interpretation of our results. Furthermore, we describe regression residuals as an alternative modeling strategy that can disentangle different variance components and allows for modeling different factor loadings, but it requires additional assumptions on the covariance structure. See also our detailed responses to the comments of Reviewer 1.

That said, it was difficult to determine which exact model was being interpreted in the results, since there is a lot of specific terminology surrounding these modeling approaches. The paper would benefit from referencing the exact Appendix Figures in the results section when discussing each set of results. Please reference these figures in your revised manuscript.

Thank you for pointing out that the results section required references to the exact models. We carefully edited the results and refer the reader to the Figures in the Appendix.

In addition to this minor edit, there are a few minor line items Reviewer 1 noted. I also have a few minor line comments. All minor comments should be addressed in the final revision.

- The OSF link did not load, so please check that it is made accessible upon publication.
- Line 449-450 “Yet, other explanations cannot be ruled out.” Can you elaborate on this a little bit more. What competing explanations cannot be ruled out?
- Line 510 has an extra or missing word. Please revise.

Thank you for the detailed suggestions. We solved all issues in the revised manuscript.

- We are sorry for the inconvenience regarding the OSF link. We double checked the link that is specified as: Anyone with the link can view this project. It should load now.
- We elaborated on other explanations for the correlation pattern of the SWLS item-effect-variables. These are in line with the item content and a reasonable explanation is sematic multidimensionality. However, the impact of more general person characteristics or method effects that interact only with the specific item content cannot be ruled out.
- We corrected all wording issues

Journal Requirements:
Please review your reference list to ensure that it is complete and correct. If you have cited papers that have been retracted, please include the rationale for doing so in the manuscript text, or remove these references and replace them with relevant current references. Any changes to the reference list should be mentioned in the rebuttal letter that accompanies your revised manuscript. If you need to cite a retracted article, indicate the article’s retracted status in the References list and also include a citation and full reference for the retraction notice.

We carefully went through the reference list and ensured that it is complete and correct.

**REVIEWERS' COMMENTS:**

**Reviewer 1:**

The authors have carefully revised the manuscript and implemented all my comments. The paper

reads very well now and I was happy to see the new appendix B.

Thank you for highlighting that our revision implemented all your former comments and for the positive feedback on the new appendix.

I have one more bigger point as a follow up on this appendix and its implications. In the results,

variance/SD of the effect variable is interpreted: “Moreover, the participants differed considerably in the size of these item effects (𝑆𝐷𝛿 = [0.39; 0.84]).”, and variance is seen as an indication of the

relevance of item-effect variables. But, the appendix shows that the variance of delta can also be big due to differences in factor loadings. For example, suppose that for two items i and j, 𝜏𝑖 = 5 ∗ 𝜏𝑗, then the standard deviation of their difference can still be considerable (4 ∗ 𝜏𝑗), even though

modeling an effect variable would not be needed. In this particular case you would find out because the correlation between delta and tau (or delta and eta, the reference item) would be 1. But, suppose that 𝜏𝑖 = 5 ∗ 𝜏𝑗 + 𝑥, where x is an item specific effect of item i. Now, the correlation would no longer be 1 and so one would start to interpret the size/SD of the effect variable, but the difference in factor loading would still influence this SD a lot. That is, even if the variance of 𝑥 would be small, the variance of delta would be big because of the big difference in factor loadings between item i and j. Because of this, I was wondering, do you really want the effect variable (delta) to be the difference, or would you rather want to regress the items on the reference item and have delta be the residual. (e.g., for 𝜏𝑖 = 5 ∗ 𝜏𝑗 + 𝑥, delta would now be 4 ∗ 𝜏𝑗 + 𝑥 but in the latter case where 𝜏𝑖 is regressed on 𝜏𝑗, delta would only be 𝑥). Sorry, I might not have thought this completely through and it might well be that I misunderstand something or there are good reasons to prefer the first over the latter, but I hope you can explain.

Thank you very much for your comments, these are very helpful for better understanding the different modeling strategies. As the reviewer points out, and as we show in Appendix B, the variance of the item-effect variables and their correlation with other variables are intertwined with differences in factor loadings. This is important for the interpretation of the size of item-effect variables as well as for the interpretation of the correlation patterns, and we draw on this in the revised manuscript.

We also refer the reader to the alternative modeling strategy of item effects as regression residuals. As the reviewer highlights, this strategy allows for different factor loadings and disentangles specific variance components. However, for identifying the additive variance components, typically the assumption is that item-effect residuals and state variables of the same construct are uncorrelated. Using the example of the reviewer (i.e., $\tau_{it}=5\tau_{1t}+x$), this means the item-effect residual can be defined as $x,$ but with the additional assumption that $Cov\left( \tau_{1t},x \right)=0.$ This is not the case in our approach, where this covariance is part of the variance of the item-effect variable. Yet, this comes at the cost that the difference variable $\delta_{it}$entangles the variance of $\tau_{1t}$ and $x$. In the example this is,

$$Var\left( \delta_{it} \right)=Var\left( 4\tau_{1t}+x \right)=4^{2}Var\left( \tau_{1t} \right)+Var\left( x \right)+2\cdot4 Cov\left( \tau_{1t},x \right).$$

Indeed, when judging the size of item effects, it is not sufficient to only draw on this variance. We carefully include this caution note in the revised manuscript and point out that the model comparison of a congeneric model with the model that includes item-effect variables is very important for investigating, the relevance of item effects in an application.

In addition, we highlight how the correlation patterns in the multi-construct model can be used for investigating the relation of the difference variables with all other variables in model. Thereby, we do not restrict specific covariances to zero, but allow for all correlations in the model -- at the cost that similarities can be obtained, but its sources cannot be disentangled (these can be prevalent either due to the state variables itself, in cases of differences in factor loadings, or due to external common causes that can in the most extreme case fully predict the differences to the states e.g., $W=\tau_{it}-\tau_{1t}=\tau_{k}-\tau_{1t}$ in Appendix B). Still, the prevalence of similarities and the patterns in the multi-construct model can be informative for substantive research to gain a better understanding of the constructs under investigation. In addition, future research may include predictors for explaining the correlation pattern.

Also I can understand that the reasons for choosing one over the other might be too extensive to

explain in the paper (I saw some alternatives being mentioned in the discussion), but then maybe it would be good to add a note when interpreting the variance or SD of the effect variables that the way these variables are defined now means that high variance of the effect variable does not

automatically mean substantial importance or relevance, in case the main explanation for the variance is differences in factor loadings (which can be investigated by analyzing the correlations?).

Indeed, thank you for pointing out that this needs to be reflected more carefully in the manuscript. We included a caution note on this and highlight the comparison with a congeneric model for investigating the relevance of item-effect variables.

At page 14 we write:

First, we investigate the relevance of item-effect variables for both constructs considering a) model fit and b) the variance of item effects. For this we a) compare the fit of a congeneric model without item-effect variables to a model with item-effect variables and b) investigate the size and significance of the variance of each item-effect variable. Note, non-neglectable variances of the item-effect variables can occur just due to differences in factor loadings. Thus, the model comparison is essential for judging the relevance of item-effect variables. Only if constant factor loadings are not sufficient for modeling differences between the items (i.e., the congeneric model does not fit the data), it is reasonable to use the more complex model with item-effect variables that entangles different sources of systematic differences between items. Then, we use the model with item-effect variables for studying the correlation pattern (i.e., the correlations of item-effect variables with each other and with the latent state variables for life satisfaction and positive affect). This provides information about the systematic of item effects within and between the constructs and can indicate possible explanations for item-effect variables.

Furthermore, we note in the interpretation of the size of the item-effect variables:

Note, that the variances entangle various differences to the reference item (e.g., differences in factor loadings, semantic multi dimensionality, method effects).

A few very minor things:

• Line 72 “potentially” should be “potential”

• Just to double check the interpretation of 𝜏𝑖𝑡 in equation 2: as I understand it, this is the true

score variable for a specific item i and specific time t, but this variable varies over persons

and situations. So, to get to a single realization of the true-score variable, one has to sample a

person and a situation within time t. If this is the correct interpretation, no changes are

needed :)

• Line 163-164 “modeling item effects as latent differences allows for estimating their expected

values and variances” here you mean expected values and variance across situations or

across people, right? Since these latent differences are stable/fixed over measurement

occasions within a person x situation combination, right?

• Line 176 and line 235-236, the term ‘systematic non-state variance’ is used. In a way, couldn’t

the item-specific method effects be considered state variables as well, in the sense that they

vary across people and situations? (in contrast to trait variables that are stable across

situations). I would think that the main contrast to the common state variable, is that these

effects are item-specific as opposed to common/shared. However, I understand that

“systematic item-specific state variance” (similar to item-specific traits) is not a very pretty

name, so I leave it up to the authors what term they pick.

• Line 303-304 “the correlation of the item-effect variables can be investigated with regard to

their size and systematic across different items and constructs.” A word seems to be missing

after “systematic” or I am not sure what a “systematic” is (systematicity?). This is also the

case for line 328 “provides information about the systematic of item effects”

Thank you for these additional comments:

- We corrected the wording issues.
- We checked our interpretation of the state variables $\tau_{it}:=E(Y_{it}|U_{t}, S_{t})$. That are defined as the conditional expectation of the item responses $Y_{it}$ given the person variable at time *t* and the situation variable at time *t.* This definition describes the possible realizations for a person at time $t$ in a specific situation at time $t$, from a set of persons and a set of situations at time $t$. In a specific application we then consider a specific person x situation combination, but in the definition LST uses a pre facto perspective for specifying the variables for all persons and all situations under investigation – as such the definition is correct, but not directly in line with the sampling process, instead it refers to a random experiment for the phenomena under investigation (see e.g., Steyer et al., 2015).
- For estimating the expected values and variance of the item-effect variables, we consider to the intraindividual differences $\delta_{it}:=\tau_{it}-\eta_{t}$ and included the assumption of stable differences over time $\delta_{it}=\delta_{is}\equiv\delta_{i}$. Thus, the expectation and variance estimates refer to all persons under investigation, assuming no variation related to the situation. We included a note on this in the revised manuscript: Thereby, modeling item effects as stable latent differences allows for estimating their expected values and variances across all persons under investigation, as well as all correlations among latent variables.
- Indeed, “systematic item-specific state variance” better describes the item-effect variables. For convenience, we now use the term “systematic item-specific variance” as this excludes random measurement error and highlights the item-specific variance component.
